# Laparoscopic transperitoneal pyelopyelostomy and ureteroureterostomy of retrocaval ureter: Report of two cases and review of the literature

**DOI:** 10.4103/0972-9941.65166

**Published:** 2010

**Authors:** Onkar Singh, Shilpi Singh Gupta, Ankur Hastir, Nand Kishore Arvind

**Affiliations:** Department of Urology, Bhopal Memorial Hospital & Research Centre, Bhopal - 462 038, India; 1 Department of Surgery, MGM Medical College & Hospital, Kamothe, Navi Mumbai - 410 209, India

**Keywords:** Retrocaval ureter, laparoscopy, pyelopyelostomy, ureteroureterostomy

## Abstract

We report two cases of retrocaval ureter that were successfully treated by a laparoscopic transperitoneal approach. Presentation of both these cases was with flank pain. Ureteroureterostomy using an intracorporeal suture technique was performed for one, and pyelopyelostomy for the other case. Operative time was 120 min and 110 min, respectively. Pyelopyelostomy was technically easier to perform than ureteroureterostomy that required an extra fourth port insertion to facilitate dissection. With increasing experience with the intracorporeal suturing laparoscopic technique of either pyelopyelostomy or ureteroureterostomy should be the first choice for retrocaval ureter.

## INTRODUCTION

Retrocaval ureter is a rare congenital anomaly that results in external ureteral compression by the inferior vena cava, and becomes symptomatic in the third or fourth decade of life.[1,2] Ureteroureterostomy and pyelopyelostomy by open surgery have been the gold standard treatment options for retrocaval ureter for many years. Although, only sporadic cases are reported in the literature, our experience with the laparoscopic approach for retrocaval ureter surgery and intracorporeal suturing has increased a lot over recent years, thus, rapidly making it the standard approach.[3] We present two cases of this uncommon anomaly treated with laparoscopic transperitoneal pyelopyelostomy and ureteroureterostomy.

## CASE REPORTS

### Case 1

**Figure 1:**
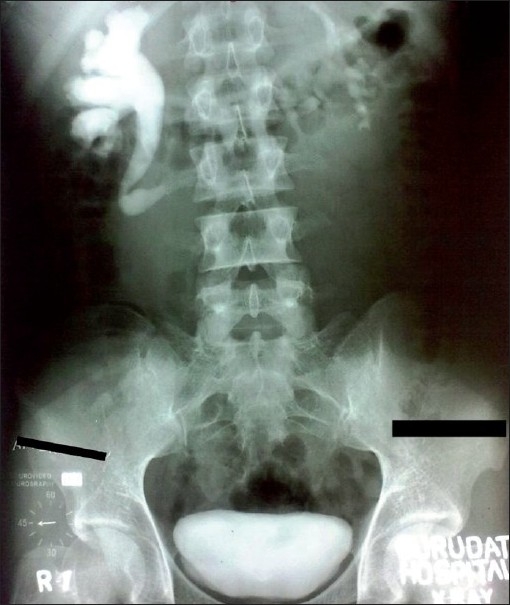
Excretory urogram showing reverse-J deformity of right proximal ureter with dilatation, associated with right hydronephrosis

**Figure 2:**
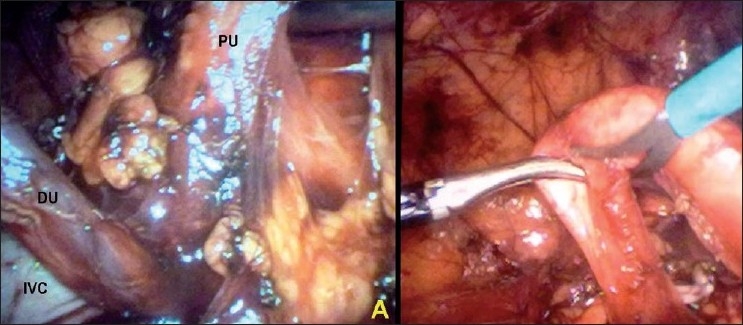
(Right) Laparoscopic transperitoneal dissection of retrocaval ureter; upper ureter (PU) can be seen as going posterior to the inferior vena cava (IVC) while distal segment of the ureter (DU) clearly seen as it emerges from posterior of IVC. (Left) The retrocaval portion of the ureter being excised.

A 21-year-old male presented with 2 months history of intermittent right flank pain. Physical examination and urine analysis were found to be normal. Haemogram and renal function tests were unremarkable. Ultrasonography (USG) revealed moderate right hydronephrosis with dilatation of proximal ureter. Excretory urography showed classical reverse-J deformity of right proximal ureter with right hydrouretero nephrosis [Figure 1] with a provisional diagnosis of retrocaval ureter. A three port approach with a primary port at 2.5 cm to the right of umbilicus, a 5 mm port midway between the primary port and right costal margin, and on right mid-clavicular line, and another 5 mm port midway between the antero-superior iliac spine and the umbilicus was used while the patient was placed in the 45° left lateral position. After reflecting the ascending colon medially, the ureter was identified in the retroperitoneum coursing posterior to the inferior vena cava (IVC), and dissected up to the lateral border of IVC [Figure 2]. Mobilization of the ureter in the interaortocaval region required another 5 mm port to be inserted at the flank. The retrocaval portion of the ureter was dissected out and appeared artetic, so excised [Figure 2]. A double J-stent was inserted laparoscopically in an antegrade manner. After spatulating the distal ureter, ureteroureterostomy was done with 4-0 polyglactin sutures. A closed suction drain was placed and kept for 48 h. The operative time was 120 min. The patient was discharged after 72 h and stent was removed after 4 weeks postoperatively. At 4 months follow up, he was asymptomatic and excretory urography did not reveal any evidence of obstruction.

### Case 2

**Figure 3:**
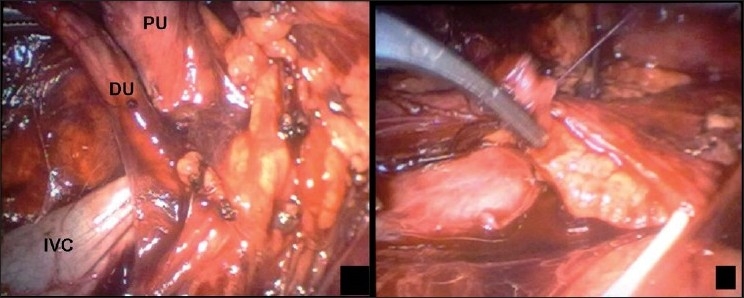
(Right) Proximal (PU) and distal (DU) segments of right ureter held up to show its course, IVC can be clearly seen as hooked up between these segments. (Left) Pyelopyelostomy being performed, double-J stent is visible with its upper end inside the pelvis.

A 24-year-old female presented with recurrent episodes of loin pain of 18 months duration. Physical examination and urine analysis was normal. Haemogram and renal function tests were unremarkable. USG showed right hydronephrosis and dilatation of proximal ureter. Excretory urography showed classical fishhook deformity of right proximal ureter just like in the previous case. Provisional diagnosis of retrocaval ureter was made and patient was elected for laparoscopic pyelopyelostomy. First, retrograde pyelography was done to see the course of the ureter. Using the three-port transperitoneal approach, the right renal pelvis was identified and dissected medially up to a point where it was just going underneath the IVC. It was also dissected free in the interaortocaval region [Figure 3]. Renal pelvis just above the ureteropelvic junction was then excised, and retrocaval portion of ureter freed completely. Pyelopyelostomy was performed antero-lateral to the IVC over a double-J stent and by using interrupted 3-0 polyglactin sutures [Figure 3]. A closed suction drain was placed and removed after 48 h. The operative time was 110 min. The patient was discharged after 72 h and stent was removed after 4 weeks postoperatively. Follow up at 5 months post-operatively with USG showed regression of hydronephrosis.

## DISCUSSION

Retrocaval ureter is a rare venous anomaly in which the right ureter courses posterior to the inferior vena cava and partially encircles it. Persistence of the subcardinal vein is the main embryologic event that underlying its development.[1] Since its first description by Hochstetter in 1893,[4] approximately 200 cases have been reported worldwide. Classically open ureteroureterostomy had been the gold standard surgical treatment for this clinical entity for many years. Over recent years, laparoscopic ureteroureterostomy and pyelopyelostomy for retrocaval ureter have been reported sporadically, that has increased our experience with the laparoscopic approach, type of surgery and intracorporeal suturing for this particular anomaly.

Baba *et al*. in 1994 were the first to perform the laparoscopic pyeloplasty for a retrocaval ureter in 9.3 h.[5] Matsuda *et al*. then performed the laparoscopic ureteroureterostomy using the five-port approach in 7.5 h.[6] In 1999, Salomon *et al*. first did the laparoscopic retroperitoneal repair of a retrocaval ureter in 4.5 h, and suggested that this was a more direct approach with greatly reduced operative time.[7] Later, Gupta *et al*. also found this approach to be safer, easier and less time-consuming (3.5 h) even using three ports.[8] Recently, Tobias-Machado *et al*. demonstrated a new technique of exteriorizing the ureter from one of the port sites and performing extracorporeal uretero-ureteral anastomosis; thus further reducing the duration of surgery to 130 min.[2] However, more recently, Nagraj *et al*. reported the shortest operative time of only 100 min with standard transperitoneal three-port laparoscopic ureteroureterostomy.[9] Simforoosh *et al*. in 2006 reported a series of six cases treated by laparoscopic pyelopyelostomy without excising the retrocaval segment.[1] Advantages mentioned by the authors included excellent outcome, minimal postoperative morbidity, short hospital stay and highly satisfactory cosmetic results, although, mean operative time was 3 h.[1] The most recent report of laparoscopic surgery of retrocaval ureter is by Chung and Gill, in which they performed dismembered pyeloplasty in 3 h.[3] Hemal *et al*. in 2008 demonstrated the feasibility of pure robotic repair, but found that apart from the ergonomic ease and simpler intracorporeal suturing, it does not appear to provide any great advantage over pure laparoscopy.[10]

The major time consuming part of laparoscopic treatment of the retrocaval ureter had been the intracorporeal anastomosis. Over the time, experience wit intracorporeal suturing has built up, leading to greatly reduced operative time from 9.3 h to less than 2 h.

In our first case, the retrocaval segment of the ureter after dissection appeared atretic. So, ureteroureterostomy was performed after excision of the atretic segment. While in the second case, retrocaval portion of the ureter was grossly normal, so we proceeded with pyelopyelostomy.

Our reports indicate that both laparoscopic transperitoneal pyelopyelostomy and ureteroureterostomy are safe and effective, and can be performed in reasonable operative time. However, pyelopyelostomy because of its technical similarity to a standard pyeloplasty with which most practicing uro-surgeons are well versed and confident while doing, is to be preferred over ureteroureterostomy, at least in today's time. Moreover, pyelopyelostomy may lead to lower stricture rate[10] and has an obvious advantage if associated pelvic stone is present. Also, relative preference may be specifically given to ureteroureterostomy if the retrocaval segment of the ureter appears grossly atretic; although Chung and Gill have performed pyeloplasty with normal urine flow at 6 months of follow-up, in a case of retrocaval ureter in which the retrocaval portion of the ureter appeared atretic.[3]

## CONCLUSION

Both laparoscopic ureteroureterostomy and pyelopyelostomy are safe and effective modes of treatment of retrocaval ureter. Laparoscopic pyelopyelostomy is technically simpler; can be performed with higher level of confidence and should be preferred if retrocaval ureter is associated with pelvic stone. Ureteroureterostomy may be specifically preferred over pyelopyelostomy when the retrocaval segment of the ureter appears grossly atretic.
